# Identification of Symptoms Prognostic of COVID-19 Severity: Multivariate Data Analysis of a Case Series in Henan Province

**DOI:** 10.2196/19636

**Published:** 2020-06-30

**Authors:** Jitian Li, Zhe Chen, Yifei Nie, Yan Ma, Qiaoyun Guo, Xiaofeng Dai

**Affiliations:** 1 Henan Provincial Orthopedic Institute Zhengzhou China; 2 Ruijin Hospital Shanghai Jiaotong University School of Medicine Shanghai China; 3 Henan Center for Disease Control and Prevention/Henan Key Laboratory of Pathogenic Microorganisms Zhengzhou China; 4 Wuxi School of Medicine Jiangnan University Wuxi China

**Keywords:** prognostic symptoms, COVID-19, severity, CVD, Henan Province

## Abstract

**Background:**

The outbreak of severe acute respiratory syndrome coronavirus 2 (SARS-CoV-2), which causes coronavirus disease (COVID-19), has been declared a global pandemic. Identifying individuals whose infection can potentially become severe is critical to control the case fatality rate of COVID-19. However, knowledge of symptoms that are prognostic of COVID-19 severity is lacking.

**Objective:**

The objective of our study was to identify symptoms prognostic of COVID-19 infection severity.

**Methods:**

We analyzed documented symptoms, including fever, cough, fatigue, expectoration, sore throat, chest distress, headache, diarrhea, rhinorrhea, stuffed nose, nausea, vomiting, muscle or joint ache, shortness of breath, and their associations with disease severity using a case series, including 655 confirmed cases from January 23 to February 5, 2020 in Henan Province, China. We also analyzed the influence of individual characteristics, including age, gender, and comorbidities, on symptoms with prognostic value.

**Results:**

Fatigue (95% CI 0.141 to 0.334, *P*<.001), expectoration (95% CI 0.107 to 0.305, *P*<.001) and stuffed nose (95% CI –0.499 to –0.082, *P*=.006) were identified as the prognostic symptoms of COVID-19 patients from the multivariate analysis. Fever occurred in 603/655 (92.1%) of the patients but was not associated with disease severity. Fatigue accounted for 184/655 (28.1%) of the patients and was linearly associated with infection severity with statistical significance. Expectoration occurred in 169/655 (25.8%) patients in the cohort and was the sole prognostic factor for patients with cardiovascular complications, including hypertension. Shortness of breath, chest distress, muscle or joint ache, and dry cough, which occurred in 33 (5%), 83 (12.7%), 78 (11.9%), and 276 (42.1%) of the 655 patients, respectively, were significantly enriched among patients classified as severe. Stuffed nose and nausea were associated with favorable disease severity, especially among male patients. More female than male patients were documented as having muscle or joint ache. Headache was most enriched in patents aged 15 to 39 years, followed by those aged 40 to 64 years, with statistical significance.

**Conclusions:**

Fatigue and expectoration are signs of severe COVID-19 infection. Shortness of breath, chest distress, muscle or joint ache, and dry cough are prevalent in severe patients. Expectoration is commonly present in older individuals and patients with cardiovascular disorders, including hypertension. Shortness of breath is prognostic of severe infection in male patients. Stuffed nose and nausea are favorable prognostic factors of severe infection, especially among male patients.

## Introduction

In early December 2019, a pneumonia of unknown etiology emerged in Wuhan, a city in China with 11 million permanent residents and 5 million recurrent residents. On December 29, 2019, the first four cases of this pneumonia were reported, all of which were linked to the Huanan Seafood Wholesale Market in Wuhan. On January 7, 2020, a novel coronavirus was identified from the bronchoalveolar lavage fluid of a patient [1] and was named severe acute respiratory syndrome coronavirus 2 (SARS-CoV-2) by the World Health Organization (WHO) [2]. SARS-CoV-2 is the seventh enveloped RNA coronavirus to be identified [3]; it is transmittable via humans and has a 3-day median incubation time [4]. This virus has rapidly spread worldwide and has become a global health threat [5]. The high binding affinity of SARS-CoV-2 to angiotensin-converting enzyme 2 enables its rapid transmission [6]. Approximately 8 million individuals were infected and over 0.4 million deaths were reported worldwide as of June 2020 [7]. The death rate varies among countries and reached as high as 27.1% in Yemen [7].

Suspected cases were identified as having “fever or respiratory symptoms” and “traveling history or contact with confirmed infections within 2 weeks” [8]. Unlike the symptoms of severe acute respiratory syndrome (SARS), where fever was the symptom in approximately 100% of infected individuals [9], fever is only observed in 87.9% of patients with COVID-19 on admission [4]; other symptoms, such as cough (67.7%) and fatigue (38.1%), also frequently occur in SARS-CoV-2–infected individuals. Despite the many reports on symptoms associated with COVID-19, little effort has been devoted to the identification of symptoms associated with its severity. This study aims to identify symptoms with prognostic value on disease severity and their correlations with individual characteristics such as age, gender, and comorbidities to aid the prognosis of COVID-19 severity.

## Methods

### Data Source

This case series was collected by the Center for Disease Control and Prevention of Henan Province (Henan CDC) from 279 hospitals in the province. It includes 655 confirmed patients with COVID-19 who showed symptoms on admission and were admitted to hospitals from January 23, 2020, to February 5, 2020, with February 5 being the last follow-up date. Oral consent was obtained from the patients. All enrolled patients were diagnosed according to the WHO interim guidance [10].

Epidemic, clinical, and severity data were obtained with data collection forms from electronic medical records as part of standard care. The information recorded included demographic data, comorbidities, symptoms, and chest computed tomography (CT) scans. The date of disease onset was defined as the day when the symptom was noted. This study was approved by the ethics commissions of the Henan CDC with a waiver of informed consent. Cardiovascular disease (CVD) is a group of disorders of the heart and blood vessels, including cerebrovascular disease, coronary heart disease, cerebrovascular disease, peripheral arterial disease, and rheumatic heart disease.

We stratified severity into three groups: light, normal, and severe. According to the Novel Coronavirus Diagnostic and Therapeutic Plan (Seventh Edition) [11], COVID-19 severity was initially divided into four types: light, normal, severe, and terminal. We merged severe and terminal patients into one group, named the severe group. The clinical symptoms of patients in the light group were mild, with no pneumonia found in imaging. Patients in the normal group had fever, respiratory tract infection, and other symptoms, with manifestation of pneumonia observable in imaging. In adults, if one of the following three conditions was satisfied, the patient was classified as severe: 1) shortness of breath, respiratory rate ≥30 times per minute; oxygen saturation ≤93% in the resting state; arterial blood sample partial pressure (PaO_2_)/oxygen concentration (FiO_2_) ≤300 millimeters of mercury. Among children, if any of the following criteria were met, the child was classified as severe: shortness of breath (≤2 months of age, respiratory rate ≥60 times/min; 2 to 12 months of age, respiratory rate ≥50 times/min; 1-5 years of age, respiratory rate ≥40 times/min; >5 years of age, respiratory rate ≥30 times/min), excluding the effects of fever and crying; oxygen saturation in the resting state is ≤92%; assisted respiration (moaning, alar fluttering, three-concave sign); cyanosis; intermittent apnea; drowsiness; convulsion; refusing to eat or difficulty in feeding; and signs of dehydration. Patients with critically severe cases satisfied the following criteria: respiratory failure that requires mechanical ventilation; shock; other organ failure that requires intensive care unit monitoring and treatment.

### Laboratory Testing

Throat or nose swab samples were collected from patients suspected of having SARS-CoV-2 infection for total RNA extraction using the respiratory sample RNA isolation kit (Shanghai BioGerm Medical Technology Co Ltd, Catalog No. ZC-HX-201-2), followed by real time reverse transcription–polymerase chain reaction (RT-PCR) using a SARS-CoV-2 nucleic acid detection kit (Shanghai BioGerm Medical Technology Co Ltd) in the biosafety level 2 lab at Henan CDC. Targeting the open reading frame (ORF1a/b), the primers and sequences were forward primer CCCTGTGGGTTTTACACTTAA, reverse primer ACGATTGTGCATCAGCTGA, and probe 5'-FAM-CCGTCTGCGGTATGTGGAAAGGTTATGG-BHQ1-3'. Targeting nucleocapsid protein, the primers and sequences were forward primer GGGGAACTTCTCCTGCTAGAAT, reverse primer CAGACATTTTGCTCTCAAGCTG, and probe 5'-FAM-TTGCTGCTGCTTGACAGATT-TAMRA-3'. Conditions for the amplifications were 50 ℃ for 10 minutes and 95 ℃ for 5 minutes, followed by 40 cycles of 95 ℃ for 10 seconds and 55 ℃ for 40 seconds. Following recommendations by the Chinese National Institute for Viral Disease Control and Prevention [12], positive and negative tests were defined as cycle threshold (C_t_) values <37 and ≥40, respectively; samples with C_t_ values between these thresholds were subjected to retesting. A case was confirmed if two targets (ORF1a or 1b, nucleocapsid protein) tested positive by real-time RT-PCR in the initial test or both tests (when a retest was needed).

### Statistical Analysis

Continuous variables were described using mean, SD, median, interquartile range (IQR), and range, and categorical variables were described by frequency and percentage. Means for continuous variables were compared using independent group tests when the data were normally distributed (Shapiro-Wilk test); otherwise, the Kruskal-Wallis H test was used (adjusted by Bonferroni correction). Proportions for categorical variables were compared using the chi-square test or Fisher exact test. The correlation of two variables was compared using Spearman rank correlation. The influencing variables for the severity of COVID-19 were analyzed using a linear regression model (forward method). All statistical analyses were performed using SPSS version 23.0 (IBM Corp). A 2-sided α less than .05 was considered statistically significant.

### IT Infrastructure

We implemented the new Public Health Emergency Management Information System of Henan Province and extracted data from the system to analyze the epidemiological characteristics of COVID-19 patients in Henan Province and develop a COVID-19 cluster statistical information template. In addition, the hospital information system, dashboards, electronic prescription system, and cloud-based medical image sharing system were used to facilitate the analysis of the clinical data of COVID-19 patients.

## Results

**Figure 1:**
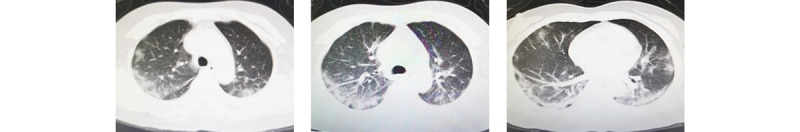
Chest computed tomographic images of a female patient aged 56 years infected with coronavirus disease. The images show ground glass opacity in both lungs on day 5 after symptom onset.

In this case series including 655 COVID-19 patients, regarding disease severity, 163 (24.9%) patients were classified as light, 420 (64.1%) as normal, and 72 (11%) as severe (Table 1). Of the infected individuals, 12/655 (1.8%) were less than 15 years of age, 265 (40.5%) were between 15 and 39 years of age, 322 (49.2%) were between 40 and 64 years of age, and 71 (8.5%) were aged 65 years or older (Table 1). Most patients were male (367/655, 56.0%). Of the 150/655 patients (22.9%) who had coexisting medical disorders, 89 (59.3%) had CVD. Given the high percentage of CVD comorbidities in this dataset, we analyzed symptoms prognostic of COVID-19 severity separately among patients with CVD, patients without CVD, and patients without comorbidities. The average number of days from illness onset to diagnosis was 5.66 (SD 3.64, range 0-32). Among the 655 patients included in this case series, 634 (96.8%) had chest CT scans, and 535 (84.4%) showed typical pneumonia features (bilateral ground glass opacities, Figure 1).

**Figure 2:**
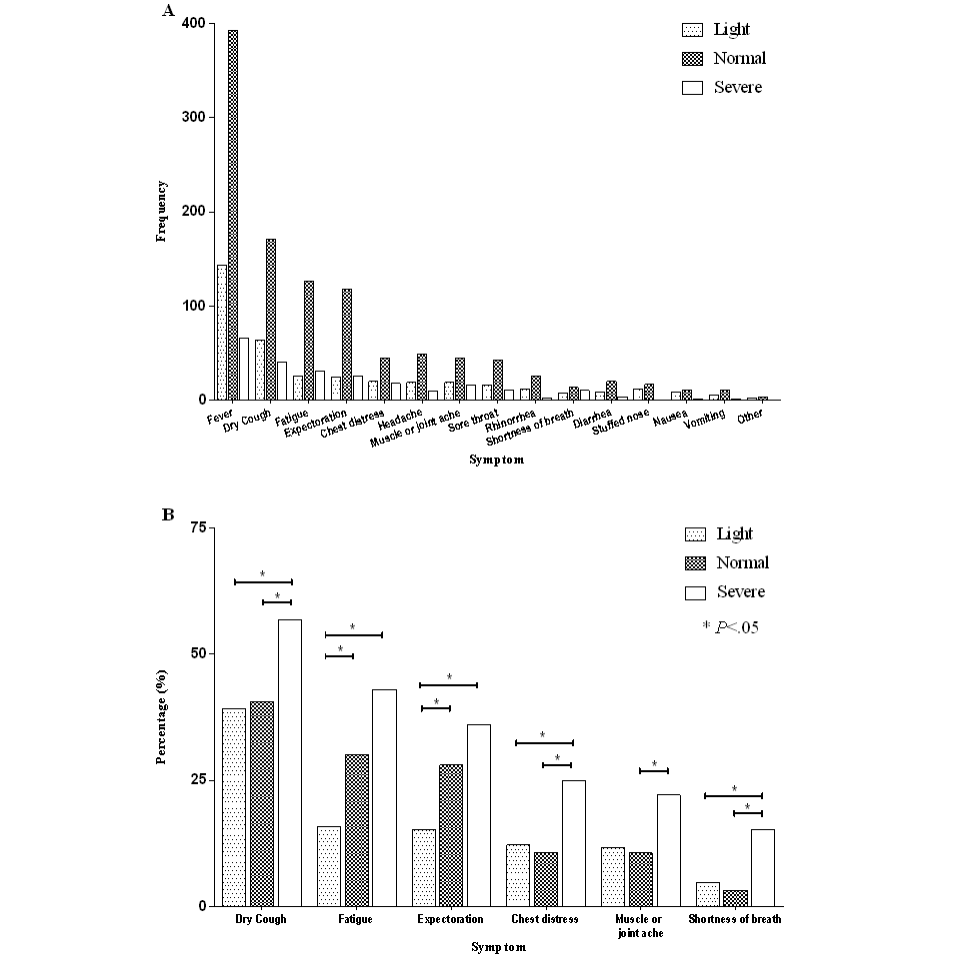
Summarized report of documented symptoms. Plots of (A) patient number of documented symptoms and (B) patient percentage of prognostic symptoms in cohorts stratified by coronavirus disease severity.

Among all documented symptoms in this case series, fever and dry cough were prevalent in all patient cohorts as stratified by COVID-19 severity. Fatigue and expectoration were enriched symptoms in the severe and normal groups (Figure 2A).

The symptom of fever occurred in 603/655 (92.1%) of the patients (Table 1) but was not associated with disease severity. A higher “highest temperature of fever” was typically associated with more severe SARS-CoV-2 infection; however, it is difficult to precisely define high and low highest temperatures of fever given the high dependence of this parameter on the time slot when it was measured and high individual heterogeneity among patients. The mean highest temperature in the light group was 38.0ºC (range 37.6-38.5ºC), that in the normal group was 38.0ºC (range 37.8-38.5ºC), and that in the severe group was 38.4ºC (range 38.0-38.9ºC) (χ^2^_2_=15.5, *P*<.001).

Other symptoms, including dry cough, fatigue, expectoration, chest distress, muscle or joint ache, shortness of breath, and multiple symptoms, all convey significant prognostic value on disease severity. Fatigue was the most prevalent symptom (184/655, 28.1%) among patients (Table 1) and could significantly (χ^2^_2_=20.8, *P*<.001) stratify and linearize patients into light, normal, and severe groups regarding clinical severity (Table 2, Figure 2A). Expectoration was observed in 169/655 (25.8%) of the patients, with significant (χ^2^_2_=14.5, *P*=.001) prognostic value on disease severity (Table 1, Table 2). Expectoration significantly (χ^2^_2_=6.6, *P*=.04) differed among patients with CVD and patients with complications other than CVD as well as patients without coexisting disorders (Multimedia Appendix 1), and it was the sole explanatory symptom included in the linear regression among CVD patients (β=0.310, 95% CI 0.042-0.579, *P*=.02, Table 3). Shortness of breath was significantly (*χ^2^*_2_=18.3, *P*<.001) enriched in the severe group (Table 2) and was observed in 33/655 (5.0%) of the patients infected with COVID-19 (Table 1). Chest distress occurred in 12.7% of this cohort (Table 1), and the sample group was significantly enriched with severely infected patients (*χ^2^*_2_=11.367, *P*=.003, Table 2). Muscle or joint ache was documented in 78/655 (11.9%) of the patients and was significantly enriched in severe patients (*χ^2^*_2_=7.7, *P*=.02, Table 2) and female patients (*χ^2^*_1_=4.5, *P*=.03, Multimedia Appendix 1). Dry cough, although it could stratify patients according to COVID-19 severity with statistical significance (*χ^2^*_2_=7.4, *P*=.03, Table 2), did not show good discrimination power (Figure 2B); this may be due to the high prevalence of dry cough among patients (276/655, 42.1%, Table 1). Headache was enriched in the 15 to 39 years age group, followed by the 40 to 64 years age group, with statistical significance (*χ^2^*_3_=11.6, *P*=.009, Multimedia Appendix 1); however, headache could not differentiate disease severity. The majority of the patients (501/655, 76.5%) reported multiple symptoms (Table 1).

**Table 1:** Documented symptoms and general clinical characteristics of 655 patients with COVID-19 (N=655).

Characteristic				n (%)
**Disease severity**				
	Light		163 (24.9)	
	Normal		420 (64.1)	
	Severe		72 (11)	
**Symptoms**				
	**Fever (ºC)**			
		<37.3	23 (3.5)	
		37.3-38.0	287 (43.8)	
		38.1-39.0	241 (36.8)	
		>39.0	37 (5.6)	
		Temperature not documented	15 (2.1)	
	Dry cough		276 (42.1)	
	Fatigue		184 (28.1)	
	Expectoration		169 (25.8)	
	Chest distress		83 (12.7)	
	Headache		80 (12.2)	
	Muscle or joint ache		78 (11.9)	
	Sore throat		70 (10.7)	
	Rhinorrhea		41 (6.3)	
	Shortness of breath		33 (5.0)	
	Diarrhea		33 (5.0)	
	Stuffed nose		30 (4.6)	
	Nausea		22 (3.4)	
	Vomiting		19 (2.9)	
	Other symptoms		7 (1.1)	
	Multiple symptoms		501 (76.5)	
**Age (years)**				
	<15		12 (1.8)	
	15-39		265 (40.5)	
	40-64		322 (49.2)	
	≥65		56 (8.5)	
**Gender**				
	Male		367 (56.0)	
	Female		288 (44.0)	
**Coexisting disorders**				
	CVD^a^		89 (59.3)	
	Disorders other than CVD		61 (40.7)	
	None		505 (77.1)	

^a^CVD: cardiovascular disease (including hypertension).

**Table 2:** Symptoms stratified by disease severity among patients with COVID-19 (N=655).

Symptom		Light (n=163), n (%)		Normal (n=420), n (%)		Severe (n=72), n (%)		Chi-square (*df*)		*P* value	
**Fever**											
	All fever		144 (88.3)		393 (93.6)		66 (91.7)		4.4 (*2*)		.11
	<37.3ºC		4 (2.9)		18 (4.6)		1 (1.6)		21.5 (*6*)^b^		.002
	37.3-38.0ºC		80 (58.4)^a^		187 (48.1)		20 (32.3)^a^				
	38.1-39.0ºC		43 (31.4)^a^		166 (42.7)		32 (51.6)^a^				
	>39.0ºC		10 (7.3)		18 (4.6)^a^		9 (14.5)^a^				
	Temperature not documented		7 (4.3)		4 (0.1)		4 (5.6)				
Dry cough		64 (39.3)		171 (40.7)		41 (56.9)^c^		7.4 (*2*)		.03	
Fatigue		26 (16.0)^c^		127 (30.2)		31 (43.1)		20.8 (*2*)		<.001	
Expectoration		25 (15.3)^c^		118 (28.1)		26 (36.1)		14.5 (*2*)		.001	
Chest distress		20 (12.3)		45 (10.7)		18 (25.0)^c^		11.4 (*2*)		.003	
Headache		20 (12.3)		49 (11.7)		10 (13.9)		0.3 (*2*)		.86	
Muscle or joint ache		19 (11.7)		45 (10.7)^a^		16 (22.2)^a^		7.7 (*2*)		.02	
Sore throat		16 (9.8)		43 (10.2)		11 (15.3)		1.8 (*2*)		.41	
Rhinorrhea		12 (7.4)		26 (6.2)		3 (4.2)		0.9 (*2*)		.65	
Shortness of breath		8 (4.9)		14 (3.3)		11 (15.3)^c^		18.3 (*2*)		<.001	
Diarrhea		9 (5.5)		20 (4.8)		4 (5.6)		0.2 (*2*)		.91	
Stuffed nose		12 (7.4)		17 (4.0)		1 (1.4)		4.8 (*2*)		.09	
Nausea		9 (5.5)		11 (2.6)		2 (2.8)		3.1 (*2*)		.21	
Vomiting		6 (3.7)		11 (2.6)		2 (2.8)		0.7 (*1*)		.78	
Other		3 (1.8)		4 (1.0)		0 (0)		1.3 (*1*)		.49	
Multiple symptoms		111 (68.1)		327 (77.9)		63 (87.50)		11.7 (*2*)		.003	

^a^Pairwise significance.

^b^Calculated with the R by C chi-square test.

^c^Significant compared with the other two groups.

Linear models were established to explain disease severity by including all patients or cohort groups stratified by age, gender, and coexisting disorder. All models constructed were significant (Table 3). Fatigue was the most frequently present symptom in these equations, followed by expectoration, and the coefficients of both symptoms were positive (Table 3). While expectoration was the sole symptom associated with patients with CVD, fatigue was linked to patients with coexisting disorders other than CVD (Table 3). Excluding patients with comorbidities did not change the variables included in the model except for slight variations in the coefficients. Stuffed nose was a negative explanatory variable in the models including all patients, individuals without comorbidities, and male patients only (Table 3). Nausea was a negative explanatory variable and played a dominant role in the equation established for male patients (Table 3).

The symptoms of highest temperature of fever (correlation 0.166, 95% CI 0.082-0.251, *P*<.001), expectoration (correlation 0.104, 95% CI 0.022-0.184, *P*=.012) and shortness of breath (correlation 0.125, 95% CI 0.001-0.041, *P*=.002) were significantly correlated with days from illness onset to diagnosis (Table 4).

**Table 3:** Linear models of clinical severity as explained by symptoms.

Stratification factor	Parameter from stepwise modeling	Regression coefficient	95% CI	*P* value	Model^a^
All	Constant	1.755	1.697 to 1.812	<.001	Y=0.237*F+0.206*E-0.291*S+1.755
	Fatigue	0.237	0.141 to 0.334	<.001	
	Expectoration	0.206	0.107 to 0.305	<.001	
	Stuffed nose	0.291	–0.499 to –0.082	.006	
Age	Constant	1.499	1.272 to 1.725	<.001	
≥65 years	Expectoration	0.656	0.253 to 1.059	.002	Y=0.460*F+0.656*E+1.499
	Fatigue	0.460	0.066 to 0.854	.02	
	Constant	1.820	1.739 to 1.900	<.001	
40-64 years	Fatigue	0.202	0.072 to 0.331	.002	Y=0.202*F+0.137*E+1.82
	Expectoration	0.137	0.001 to 0.274	.048	
	Constant	1.789	1.710 to 1.868	<.001	
15-39 years	Fatigue	0.166	0.008 to 0.324	.04	Y=0.166*F+1.789
	Constant	1.20	0.918 to 1.482	<.001	
<15 years	Fatigue	0.800	0.110 to 1.490	.03	Y=0.8*F+1.2
Gender	Constant	1.783	1.706 to 1.860	<.001	
Male gender	Fatigue	0.219	0.091 to 0.346	.001	Y=0.219*F+0.212*E-0.513*N-0.343*S+0.276*B+1.783
	Expectoration	0.212	0.083 to 0.340	.001	
	Nausea	–0.513	–0.880 to –0.147	.006	
	Stuffed nose	–0.343	–0.602 to –0.084	.01	
	Shortness of breath	0.276	0.027 to 0.525	.03	
	Constant	1.718	1.632 to 1.803	<.001	
Female gender	Fatigue	0.252	0.100 to 0.404	.001	Y=0.252*F+0.164*E+1.718
	Expectoration	0.164	0.006 to 0.322	.04	
Coexisting disorder	Constant	1.864	1.727 to 2.000	<.001	
Coexisting CVD^b^	Expectoration	0.310	0.042 to 0.579	.02	Y=0.31*E+1.864
	Constant	1.725	1.548 to 1.902	<.001	
Coexisting disorder other than CVD	Fatigue	0.418	0.116 to 0.720	.008	Y=0.418*F+1.725
	Constant	1.736	1.672 to1.800	<.001	
No coexisting disorder	Fatigue	0.264	0.152 to 0.376	<.001	Y=0.264*F+0.227*E-0.314*S+1.736
	Expectoration	0.227	0.111 to 0.343	<.001	
	Stuffed nose	–0.314	–0.548 to –0.080	.009	

^a^B: shortness of breath. E: expectoration. F: fatigue. N: nausea. S: stuffed nose.

^b^CVD: cardiovascular disease.

**Table 4:** Spearman correlation analysis of symptoms and days from illness onset to diagnosis.

Symptoms	Days from illness onset to diagnosis		
	Correlation	95% CI	*P* value
Fever	–0.039	–0.119 to 0.065	.35
Highest temperature of fever	0.166^a^	0.082 to 0.251	<.001
Temperature <37.3ºC	–0.130	–0.492 to 0.306	.55
Temperature 37.3-38.0ºC	0.006	–0.116 to 0.124	.91
Temperature 38.1-39.0ºC	0.009	–0.121 to 0.139	.89
Temperature >39.0ºC	0.109	–0.190 to 0.403	.52
Dry cough	0.058	–0.021 to 0.132	.15
Fatigue	0.023	–0.067 to 0.101	.58
Expectoration	0.104^b^	0.022 to 0.184	.012
Chest distress	0.070	–0.011 to 0.157	.09
Headache	–0.037	–0.117 to 0.044	.37
Muscle or joint ache	0.013	–0.069 to 0.093	.76
Sore throat	0.008	–0.071 t 0.080	.85
Rhinorrhea	–0.041	–0.114 to 0.033	.33
Shortness of breath	0.125^a^	0.001 to 0.041	.002
Diarrhea	–0.004	–0.086 to 0.087	.92
Stuffed nose	–0.065	–0.152 to 0.033	.12
Nausea	0.018	–0.072 to 0.103	.66
Vomiting	–0.032	–0.131 to 0.063	.43
Multiple symptoms	0.079	–0.002 to 0.158	.056

^a^*P*<.01 (two-tailed).

^b^*P*<.05 (two-tailed).

## Discussion

### Principal Findings

The main findings of this study were that fatigue and expectoration are signs of severe COVID-19 infection and that stuffed nose and nausea are favorable prognostic factors of disease severity.

Two symptoms, fatigue and expectoration, showed linear associations with COVID-19 severity (Figure 2B). Four symptoms, namely shortness of breath, chest distress, muscle or joint ache, and dry cough, were more commonly present in severe patients. Thus, if more patients are documented with these symptoms, more resources for intensive medical care should be administrated.

These prognostic symptoms were interconnected with gender, CVD, and age. The enrichment of shortness of breath in severe patients was the most evident, especially among male patients; this suggests its feasibility as a sign (with relatively low type I error) for intensive care among confirmed cases. Stuffed nose and nausea were associated with less severe COVID-19, especially among male patients. Expectoration was significantly associated with CVD complications, suggesting a correlation between CVD and lower respiratory tract infection. An age of 40 years was shown to be a breakpoint for symptoms prognostic of disease severity. While fatigue conveyed prognostic value for all age groups, expectoration showed significance when the patients’ age exceeded 40 years and dominated the model when their age exceeded 65 years. This may be due to the increased likelihood of developing CVD with increasing age and explainable by the strong association between expectoration and CVD comorbidity. As a clinical suggestion, individuals concomitantly having these characteristics and symptoms should be suspected for infection and given immediate quarantine and potential intensive medical care during the COVID-19 epidemic.

It is known that immune response to virus infection plays a vital role in the inflammation involved in heart diseases such as myocarditis, atherosclerosis, and cardiac insufficiency, and it constitutes the pathogenesis of cardiac disorder in humans [13]. Shortness of breath is a typical sign of heart and lung conditions, and fever is a symptom of stimulated immune response to infection. It was observed from our study that expectoration was associated with CVD and male gender; expectoration, shortness of breath, and fever were significantly correlated with days from illness onset to diagnosis. Therefore, we expect that patients with comorbidities, especially CVD, would experience exacerbated COVID-19 severity.

It is worth mentioning that symptoms prognostic of COVID-19 severity differ from symptoms for early COVID-19 diagnosis. While fever and dry cough were the most prevalent symptoms among infected individuals, fatigue, expectoration, and chest distress conveyed prognostic value on disease severity. This can be explained by the small percentage of patients represented in the whole case series (72, 11%) who could not be predicted from prevalent symptoms.

Although many case series including higher numbers of cases than that in this study have been reported [4,14-16], given the rapid changing global situation of COVID-19 and the sharp rise in the number of infected cases during the past few months, relatively little research has been devoted to studying the prognostic value of symptoms on severity. The sole relevant study was reported by Dong et al [17], who analyzed data from 663 patients. Compared with Dong’s study, which analyzed two symptoms (expectoration and muscle ache), one laboratory test index (albumin), and one patient characteristic (gender), our study concentrates on a more focused and complete list or characteristics that encompasses 14 symptoms. However, this study is limited by its high dependence on the accuracy and completeness of the symptoms recorded for each patient.

### Conclusions

Our study provides a statistical analysis of documented symptoms of 655 confirmed COVID-19 patients from Henan Province to aid SARS-CoV-2 diagnosis and prognosis. We conclude that fatigue and expectoration are the most important symptoms prognostic of severe COVID-19, and gender, age, and CVD comorbidity are factors associated with these symptoms; muscle or joint ache commonly occurs in female patients, and younger patients are likely to develop headache; and high temperature in fever, expectoration, and shortness of breath are typically associated with delayed diagnosis.

## Appendix

Multimedia Appendix 1 Symptoms stratified by CVD disorder, gender, and age in a case series of COVID-19 patients from Henan Province (N=655).

## Abbreviations

COVID-19: coronavirus disease
C_t_: cycle threshold
CT: computed tomography
CVD: cardiovascular disease
Henan CD: Center for Disease Control and Prevention of Henan Province
IQR: interquartile range
RT-PCR: real time reverse transcription–polymerase chain reaction
SARS: severe acute respiratory syndrome
SARS-CoV-2: severe acute respiratory syndrome coronavirus 2
WHO: World Health Organization
