# Supplementary material for: Identification of Symptoms Prognostic of COVID-19 Severity: Multivariate Data Analysis of a Case Series in Henan Province
Source: J Med Internet Res. 2020 Jun 30;22(6):e19636. doi: 10.2196/19636 (PMC7332230; doi:10.2196/19636)
Supplement: Multimedia Appendix 1 [file jmir_v22i6e19636_app1.docx]

Multimedia Appendix 1. Symptoms stratified by CVD disorder, gender, and age in a case series of COVID-19 patients from Henan Province (N=655).

| Symptoms | | Coexisting disorder | | | | | Gender | | | | Age (years) | | | | | |
| --- | --- | --- | --- | --- | --- | --- | --- | --- | --- | --- | --- | --- | --- | --- | --- | --- |
|  |  | CVD, n (%) | Non-CVD, n (%) | None, n (%) | Chi-square (*df*) | *P* value | Male, n (%) | Female, n (%) | Chi-square (*df*) | *P* value | ≥65, n (%) | 40-64, n (%) | 15-39, n (%) | <15, n (%) | Chi-square (*df*) | *P* value |
| **Fever** | |  |  |  |  |  |  |  |  |  |  |  |  |  |  |  |
|  | All fever | 82 (92.1) | 54 (88.5) | 467 (92.5) | 12 (*2*) | .56 | 343 (93.5) | 260 (90.3) | 2.2 (*1*) | .14 | 47 (83.9) | 299 (92.9) | 247 (93.2) | 10 (83.3) | 6.9 | .07 |
|  | <37.3 | 3 (3.6) | 1 (1.9) | 19 (4.2) | 2.8 (*6*)^a^ | .84 | 10 (3.1) | 13 (5.0) | 4.5 (*3*)^a^ | .22 | 0 | 13 (4.4) | 9 (3.8) | 1 (10.0) | 9.3 (*9*)^a^ | .41 |
|  | 37.3-38.0 | 37 (44.6) | 26 (50.0) | 224 (49.4) |  |  | 151 (46.2) | 136 (52.1) |  |  | 27 (56.3) | 142 (48.1) | 114 (48.5) | 4 (40.0) |  |  |
|  | 38.1-39.0 | 35 (42.2) | 23 (44.2) | 183 (40.4) |  |  | 145 (44.3) | 96 (36.8) |  |  | 20 (41.7) | 122 (41.4) | 94 (40.0) | 5 (50.0) |  |  |
|  | >39.0 | 8 (9.6) | 2 (3.8) | 27 (6.0) |  |  | 21 (6.4) | 16 (6.1) |  |  | 1 (2.1) | 18 (6.1) | 18 (7.7) | 0 |  |  |
|  | NA | 6 (6.7) | 9 (14.8) | 52 (10.3) |  |  | 40 (10.9) | 27 (10.4) |  |  | 8 (14.3) | 27 (8.4) | 30 (11.3) | 2 (16.7) |  |  |
| Dry cough | | 41 (46.1) | 29 (47.5) | 206 (40.8) | 1.7 (*2*) | .43 | 158 (43.1) | 118 (41.0) | 0.3 (*1*) | .59 | 25 (44.6) | 135 (41.9) | 110 (41.5) | 6 (50.0) | 0.5 (*3*) | .92 |
| Fatigue | | 30 (33.7) | 21 (34.4) | 133 (26.3) | 3.4 (*2*) | .19 | 108 (29.4) | 76 (26.4) | 0.7 (*1*) | .39 | 15 (26.8) | 101 (31.4) | 66 (24.9) | 2 (16.7) | 3.9 (*3*) | .28 |
| Expectoration | | 23 (25.8) | 24 (39.3)^b^ | 122 (24.2)^b^ | 6.6 (*2*) | .04 | 101 (27.5) | 68 (23.6) | 1.3 (*1*) | .26 | 14 (25.0) | 85 (26.4) | 70 (26.4) | 0 | 4.3 (*3*) | .23 |
| Chest distress | | 12 (13.5) | 10 (16.4) | 61 (12.1) | 1.0 (*2*) | .61 | 44 (12.0) | 39 (13.5) | 0.4 (*1*) | .55 | 11 (19.6) | 40 (12.4) | 31 (11.7) | 1 (8.3) | 2.9 (*3*) | .41 |
| Headache | | 5 (5.6) | 5 (8.2) | 69 (13.7) | 5.6 (*2*) | .06 | 42 (11.4) | 37 (12.8) | 0.3 (*1*) | .58 | 2 (3.6) | 33 (10.2) | 44 (16.6) | 0 | 11.6 (*3*) | .009 |
| Muscle or joint ache | | 15 (16.9) | 6 (9.8) | 59 (11.7) | 2.2 (*2*) | .33 | 36 (9.8) | 44 (15.3) | 4.5 (*1*) | .03 | 5 (8.9) | 40 (12.4) | 35 (13.2) | 0 | 2.5 (*3*) | 0.48 |
| Sore throat | | 9 (10.1) | 5 (8.2) | 56 (11.1) | 0.5 (*2*) | .77 | 37 (10.1) | 33 (11.5) | 0.3 (*1*) | .57 | 3 (5.4) | 36 (11.2) | 31 (11.7) | 0 | 3.5 (*3*) | .33 |
| Rhinorrhea | | 2 (2.2) | 5 (8.2) | 34 (6.7) | 3.0 (*2*) | .22 | 26 (7.1) | 15 (5.2) | 1.0 (*1*) | .33 | 3 (5.4) | 14 (4.3) | 22 (8.3) | 2 (16.7) | 6.4 | .08 |
| Shortness of breath | | 6 (6.7) | 4 (6.6) | 23 (4.6) | 1.5 | .49 | 21 (5.7) | 12 (4.2) | 0.8 (*1*) | .37 | 3 (5.4) | 18 (5.6) | 11 (4.2) | 1 (8.3) | 1.6 | 0.60 |
| Diarrhea | | 8 (9.0) | 4 (6.6) | 21 (4.2) | 4.2 | .11 | 22 (6.0) | 11 (3.8) | 1.6 (*1*) | .21 | 3 (5.4) | 18 (5.6) | 11 (4.2) | 1 (8.3) | 1.6 | .60 |
| Stuffed nose | | 3 (3.4) | 3 (4.9) | 24 (4.8) | 0.3 | .90 | 19 (5.2) | 11 (3.8) | 0.7 (*1*) | .41 | 5 (8.9) | 13 (4.0) | 11 (4.2) | 1 (8.3) | 3.8 | .27 |
| Nausea | | 4 (4.5) | 1 (1.6) | 17 (3.4) | 0.8 | .69 | 9 (2.5) | 13 (4.5) | 2.1 (*1*) | .15 | 2 (3.6) | 14 (4.3) | 6 (2.3) | 0 | 2.0 | .54 |
| Vomiting | | 4 (4.5) | 1 (1.6) | 14 (2.8) | 1.2 | .60 | 12 (3.3) | 7 (2.4) | 0.4 (*1*) | .53 | 4 (7.1) | 10 (3.1) | 4 (1.5) | 1 (8.3) | 7.2 | .0502 |
| Other | | 2 (2.2) | 0 | 5 (1.0) | 1.6 | .36 | 2 (0.5) | 5 (1.7) | 1.2 (*1*) | .28 | 3 (5.4)^b^ | 3 (0.9) | 1 (0.4)^b^ | 0 | 7.8 | .04 |
| Multiple symptoms | | 73 (82.0) | 50 (82.0) | 378 (74.9) | 3.3 (*2*) | .19 | 283 (77.1) | 218 (75.7) | 0.2 (*1*) | .67 | 41 (73.2) | 249 (77.3) | 205 (77.4) | 6 (50.0) | 5.3 (*3*) | .15 |

^a^Calculated by R by C chi-square test.

^a^Pairwise significance.
